# Supplementary material for: Schooling amidst a pandemic in the United States: Parents’ perceptions about reopening schools and anticipated challenges during COVID-19
Source: PLoS One. 2022 Aug 10;17(8):e0268427. doi: 10.1371/journal.pone.0268427 (PMC9365177; doi:10.1371/journal.pone.0268427)
Supplement: S1 Table — (DOCX) [file pone.0268427.s001.docx]

**S1 Table.** Survey questions included in this analysis

| At this time, are you planning to return your child to school for the 2020-2021 school year | 1=Yes 0=No 2=Unsure 3=It depends on what plans the school district makes 4=Prefer not to say |
| --- | --- |
| Which of the following statements are true when thinking of the 2020-2021 school year? Select all that apply | 1= My child cannot carry out distance learning from home due to lack of child care 2= Carrying out distance learning from home will place an extremely difficult burden on my family 3= My child will require supervision at school before the school day begins 4= My child will require supervision at school after the school day ends 5= My child will rely on the school/district for meals and receiving enough food 6= My child will rely on school district transportation (school bus) 7= None of these apply to me 8= Prefer not to say |
| Do you have access to necessary technology resources (e.g., Laptop, High-speed internet) to support your child's remote learning, if required ? | 1=Yes 2=No 3=Didn't need to use any technology 98=Prefer not to say |
| Which of the following statements best describes your overall feeling about returning your child to school? | 1 = My child will only return if there is a vaccine available  2 = My child will only return if social distancing, handwashing, face-covering guidelines, and temperature checks are encouraged  3 = My child will only return if social distancing, handwashing, face-covering guidelines, and temperature checks are enforced  4 = My current financial situation will force me to send my child back to school because I/we work and cannot afford childcare  5 = My child will return based on whatever safeguards the school district has in place  6 = Prefer not to say  7 = Other |
| Many factors are important to consider when we reopen schools. Please select your **top five** priorities regarding health safety practices if schools were to open for in-person classes this fall | 1=COVID-19 vaccine availability 2=COVID-19 testing and COVID-19 antibody testing availability 3=Availability of masks/face-coverings for everyone 4= Requirement for teachers/staff to wear a mask/face-covering 5= Requirement for students to wear a mask/face-covering 6= Regularly scheduled, adult supervised hand-washing 7= Staff are trained on CDC guidance to reduce likelihood of COVID-19 transmission 8= Temperature screening for students and staff each day 9=Hand sanitizer is provided in each classroom and is frequently used 10=Cafeteria seating is spaced for proper social distancing and food in individually packaged 11= Staggering schedules to reduce crowding 12= Limiting classroom seating to maintain social distancing 13= Enhanced cleaning of surfaces in the schools. 14= Other |
