# Supplementary material for: Schooling amidst a pandemic in the United States: Parents’ perceptions about reopening schools and anticipated challenges during COVID-19
Source: PLoS One. 2022 Aug 10;17(8):e0268427. doi: 10.1371/journal.pone.0268427 (PMC9365177; doi:10.1371/journal.pone.0268427)
Supplement: S2 Table — (DOCX) [file pone.0268427.s002.docx]

**S2 Table.** Parents' plans to return their children to school in the 2020-2021 academic year

|  | **Parents plans to return their children to school during the 2020-2021 academic year** | | |  | |  |
| --- | --- | --- | --- | --- | --- | --- |
|  | **Yes**  **N (%)** | **No**  **N (%)** | **Depends^a^**  **N (%)** | **Total**  **N (%)** | | **p-value** |
| **Age** | | | | | | |
| 18-24 | 25 (10.04) | 10 (6.25) | 7 (7.61) | 42 (8.38) | |  |
| 25-34 | 101 (40.56) | 60 (37.50) | 31 (33.70) | 192 (38.32) | |  |
| 35-44 | 82 (32.93) | 59 (36.88) | 28 (30.43) | 169 (33.73) | |  |
| 45-54 | 29 (11.65) | 24 (15.00) | 17 (18.48) | 70 (13.97) | |  |
| 55+ | 12 (4.82) | 7 (4.38) | 9 (9.78) | 28 (5.59) | | 0.307 |
| **Gender** | | | | | | |
| Female | 113 (45.56) | 95 (59.38) | 53 (58.24) | 261 (52.30) | |  |
| Male | 135 (54.44) | 65 (40.63) | 38 (41.76) | 238 (47.70) | | 0.011 |
| **Race** | | | | | | |
| White | 115 (46.18) | 64 (40.00) | 33 (35.87) | 212 (42.32) | |  |
| African American | 58 (23.29) | 40 (25.00) | 25 (27.17) | 123 (24.55) | |  |
| Latino | 54 (21.69) | 48 (30.00) | 30 (32.61) | 132 (26.35) | |  |
| Other | 22 (8.84) | 8 (5.00) | 4 (4.35) | 34 (6.79) | | 0.136 |
| **Education** | | | | | | |
| HS or less | 36 (14.46) | 32 (20.00) | 18 (19.78) | 86 (17.20) | |  |
| Associate degree | 27 (10.84) | 24 (15.00) | 18 (19.78) | 69 (13.80) | |  |
| Some college but no degree | 37 (14.86) | 31 (19.38) | 13 (14.29) | 81 (16.20) | |  |
| Bachelors | 89 (35.74) | 38 (23.75) | 26 (28.57) | 153 (30.60) | |  |
| Graduate | 60 (24.10) | 35 (21.88) | 16 (17.58) | 111 (22.20) | | 0.088 |
| **Income** | | | | | | |
| <$20,000 | 25 (10.04) | 22 (13.84) | 9 (9.78) | 56 (11.20) | |  |
| $20,000 to $39,999 | 36 (14.46) | 24 (15.09) | 17 (18.48) | 77 (15.40) | |  |
| $40,000 to $69,999 | 49 (19.68) | 39 (24.53) | 30 (32.61) | 118 (23.60) | |  |
| $70,000 to $99,999 | 63 (25.30) | 32 (20.13) | 16 (17.39) | 111 (22.20) | |  |
| >100K+ | 76 (30.52) | 42 (26.42) | 20 (21.74) | 138 (27.60) | | 0.182 |
| **School level of child** | | | | | | |
| Daycare | 25 (10.04) | 22 (13.75) | 10 (10.87) | 57 (11.38) | |  |
| Elementary & Middle | 114 (45.78) | 83 (51.88) | 42 (45.65) | 239 (47.70) | |  |
| High School | 53 (21.29) | 30 (18.75) | 27 (29.35) | 110 (21.96) | |  |
| More than one school type | 57 (22.89) | 25 (15.63) | 13 (14.13) | 95 (18.96) | | 0.160 |
| **School type of child** | | | | | |  |
| Public | 160 (65.31) | 106 (67.95) | 69 (75.00) | | 335 (76.83) |  |
| Private or religious | 85 (34.69) | 50 (32.05) | 23 (25.00) | | 101(23.17) | 0.236 |

^a^Depends on what plans the district makes
