# Supplementary material for: Schooling amidst a pandemic in the United States: Parents’ perceptions about reopening schools and anticipated challenges during COVID-19
Source: PLoS One. 2022 Aug 10;17(8):e0268427. doi: 10.1371/journal.pone.0268427 (PMC9365177; doi:10.1371/journal.pone.0268427)
Supplement: S3 Table — (DOCX) [file pone.0268427.s003.docx]

**S3 Table.** Parents' access to technology for remote learning during the 2020-2021 academic year

|  | **Access to tech for remote learning** | | |  |
| --- | --- | --- | --- | --- |
|  | Yes  n (%) | No  n (%) | Don't need to use  n (%) | p-value |
| **Age** | | | | |
| 18-24 | 37 (8.67) | 4 (6.25) | 2 (13.33) |  |
| 25-34 | 154 (36.07) | 31 (48.44) | 9 (60.00) |  |
| 35-44 | 152 (35.6) | 17 (26.56) | 2 (13.33) |  |
| 45-54 | 64 (14.99) | 6 (9.38) | 1 (6.67) |  |
| 55+ | 20 (4.68) | 6 (9.38) | 1 (6.67) | 0.135 |
| **Gender** | | | | |
| Female | 228 (53.4) | 30 (47.62) | 8 (53.33) |  |
| Male | 199 (46.6) | 33 (52.38) | 7 (46.67) | 0.692 |
| **Race** | | | | |
| White | 180 (42.15) | 27 (42.19) | 7 (46.67) |  |
| African American | 105 (24.59) | 18 (28.13) | 3 (20.00) |  |
| Latino | 114 (26.70) | 13 (20.31) | 4 (26.67) |  |
| Other | 28 (6.56) | 6 (9.38) | 1 (6.67) | 0.918 |
| **Education** | | | | |
| HS or less | 77 (18.03) | 8 (12.50) | 2 (13.33) |  |
| Associate degree | 59 (13.82) | 10 (15.63) | 1 (6.67) |  |
| Some college but no degree | 70 (16.39) | 8 (12.5) | 5 (33.33) |  |
| Bachelors | 129 (30.21) | 20 (31.25) | 4 (26.67) |  |
| Graduate | 92 (21.55) | 18 (28.13) | 3 (20.00) | 0.620 |
| **Income** | | | | |
| <$20,000 | 49 (11.50) | 9 (14.06) | 0 (0.00) |  |
| $20,000 to $39,999 | 65 (15.26) | 8 (12.50) | 4 (26.67) |  |
| $40,000 to $69,999 | 100 (23.47) | 15 (23.44) | 7 (46.67) |  |
| $70,000 to $99,999 | 98 (23.00) | 12 (18.75) | 0 (0.00) |  |
| >100K+ | 114 (26.76) | 20 (31.25) | 4 (26.67) | 0.192 |
| **School level of child** | | | | |
| Daycare | 40 (9.37) | 11 (17.19) | 6 (40.00) |  |
| Elementary & Middle | 208 (48.71) | 29 (45.31) | 6 (40.00) |  |
| High School | 100 (23.42) | 10 (15.63) | 1 (6.67) |  |
| More than one school type | 79 (18.50) | 14 (21.88) | 2 (13.33) | 0.005^a^ |
| **School type of child** | | | | |
| Public | 299 (71.19) | 34 (53.97) | 7 (46.67) |  |
| Private or religious | 121 (28.81) | 29 (46.03) | 8 (53.33) | 0.004^a^ |

^a^statistically significant at p-value=0.050
